# Lactate Changes Quantified by fMRS: A Meta‐Analysis

**DOI:** 10.1002/nbm.70295

**Published:** 2026-05-04

**Authors:** Luca Cairone, Maria Guidi, Matteo Mancini, Federico Giove

**Affiliations:** ^1^ Università degli Studi di Roma La Sapienza Rome Italy; ^2^ Centro Ricerche Enrico Fermi Rome Italy; ^3^ Cardiff University Brain Research Imaging Centre (CUBRIC) Cardiff University Cardiff UK; ^4^ Fondazione Santa Lucia IRCCS Rome Italy

**Keywords:** brain metabolism, fMRS, functional magnetic resonance spectroscopy, lactate

## Abstract

Over the past 15 years, the number of studies employing functional magnetic resonance spectroscopy (fMRS) has tripled, driven in part by improvements in scanner performance and a growing interest in the investigation of metabolic dynamics associated with the modulation of brain activity. Higher static magnetic fields, enhancements in gradient strength and stability, more sensitive RF coil designs, and refinements in quantification approaches have considerably increased spectral quality and temporal resolution. Together, these developments have further strengthened the unique ability of fMRS to monitor in vivo metabolic changes. Given its role in oxidative metabolism and its relevance in brain energetics, lactate (Lac) is one of the most studied metabolites, following the most important excitatory (glutamate [Glu]) and inhibitory (GABA) neurotransmitters. This meta‐analysis aims to obtain an estimate of mean Lac changes in the healthy human brain during a task, by grouping together the papers published to date according to magnetic field strength, spectroscopic sequence echo time, and task design. Across all included studies, an increase in Lac concentration during stimulation is reported by all the papers included in this meta‐analysis, with a statistically significant increase between the stimulation and rest periods of 22%. Studies using visual stimuli reported a significantly higher increase of Lac compared to studies employing motor or cognitive tasks. No significant differences emerged between studies at different magnetic field strengths or echo times. This meta‐analysis, however, revealed a substantial methodological heterogeneity, highlighting the need for greater standardization in fMRS methodologies.

## Graphical abstract

A systematic meta‐analysis of fMRS studies investigating brain lactate in healthy human subjects was conducted, with experiments categorized according to magnetic field strength, spectroscopic echo time, and task design. Across all included studies, lactate concentration was consistently reported to increase during functional stimulation, showing an overall statistically significant rise of 22% when compared with resting conditions.

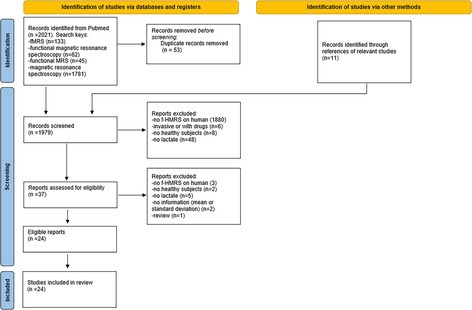

**Abbreviations**
ACC: anterior cingulate cortex
ANLSH: astrocyte‐neuron lactate shuttle
BOLD: blood oxygenation level‐dependent
CBF: cerebral blood flow
CI: confidence interval
CMRGlc: cerebral metabolic rate of glucose
CMRO_2_
: cerebral metabolic rate of oxygen
fMRS: functional magnetic resonance spectroscopy
GABA: γ‐aminobutyric acid
Glu: glutamate
IFG: inferior frontal gyrus
Lac: lactate
NALSH: neuron‐to‐astrocytes lactate shuttle
NBR: negative BOLD response
SD: standard deviation
SEM: standard error of the mean
SNR: signal‐to‐noise ratio
VOI: voxel of interest

## 1 Introduction

### 1.1 Background

Functional magnetic resonance spectroscopy (fMRS) combines the functionality of MR spectroscopy with that of fMRI, enabling the dynamic quantification of metabolite concentrations associated with cognitive or stimulation states. In fMRS, the metabolic content is assessed noninvasively in a brain area that is functionally engaged during the acquisition, that is, a region in which typically a change in the blood oxygenation level‐dependent (BOLD) signal occurs. Although the BOLD signal reflects hemodynamic and metabolic responses and is thus linked to cerebral metabolism [1], it does not provide a direct or quantitative assessment of the latter. Conversely, a comprehensive understanding of the physiological meaning of the BOLD signal requires a detailed characterization of the metabolic and vascular processes that support neural activation, including changes in cerebral blood flow, blood volume, oxygen consumption, and the associated shifts in energy metabolism. Combining multiple MRI and MRS techniques with modeling is essential for identifying specific relationships between changes in metabolic concentrations and variations in the BOLD signal [2], as well as for interpreting the metabolic contributions and the role of oxidative metabolism in the generation of BOLD contrast [3].

In more general terms, fMRS is crucial for the in vivo assessment of metabolic dynamics in humans. Since the pioneering study by Prichard and colleagues [4] in 1991, which focused on lactate, interest in fMRS has steadily grown. However, persistent technical challenges have limited its widespread application. Functional MRS studies employing visual stimulation are relatively common [2, 5, 6], but numerous investigations have also explored responses to painful stimulations [7, 8, 9], learning tasks [10, 11, 12], and motor tasks [13, 14, 15], across magnetic field strengths ranging from 1.5 to 9.4 T.

Although it is well known that many brain diseases, including Alzheimer's disease [16, 17], schizophrenia [18, 19], and epilepsy [20, 21], are associated with metabolic abnormalities, most functional spectroscopy studies are based on healthy subjects. In fact, only a few studies have applied fMRS to clinical populations, including schizophrenia [22, 23, 24], major depressive disorder [22], bipolar disorder [24], Parkinson's disease [25], obsessive‐compulsive disorder [26], migraine [27, 28], and Alzheimer's disease [29]. The predominance of fMRS studies in healthy subjects reflects the technical complexity of the method and the challenges of obtaining high‐quality spectra in clinical populations.

The key players of metabolic response to stimuli are the metabolites involved in energetic metabolism, including the major inhibitory (GABA) and excitatory (glutamate) neurotransmitters. Both GABA and glutamate are involved in multiple metabolic cycles relevant to neurotransmission and neurochemical response to stimulation [30, 31]. Experimentally, a significant increase in glutamate and nonsignificant variations in GABA under stimulation are typically reported [32].

Recently, the first fMRS studies of areas showing a negative BOLD response (NBR) have been published [33, 34], reporting a decrease in glutamate concentration. Such studies remain relatively scarce, partly due to the challenges of reliably inducing a homogeneous and sustained NBR suitable for fMRS acquisition [35, 36]. Although most attention has focused on glutamate, owing to its dual role as a neurotransmitter and a metabolic intermediate in oxidative metabolism, lactate is historically a metabolite of particular interest for fMRS; understanding lactate dynamics in the brain is important because lactate serves not only as a marker of ongoing energy metabolism but also as a key signaling molecule involved in neuron–glia interactions [37, 38], neurotransmission [39], and the coupling between neural activity and energy supply [40].

### 1.2 The Role of Lactate

Lactic acid, or its conjugated base lactate, is a molecule produced mainly during anaerobic glycolysis, that is, in the absence or scarcity of oxygen, although it is also produced under aerobic conditions in the brain. Lactate is the end product of the conversion of pyruvate (Pyr) by the enzyme lactate dehydrogenase, which occurs when there is an imbalance due to a greater production than consumption of Pyr in the cytosol. Before the development of the Pellerin–Magistretti hypothesis of the astrocyte‐neuron lactate shuttle (ANLSH) [41], lactate was thought to be a waste product, especially after the observations of the uncoupling between CBF, CMRO_2_, and cerebral metabolic rate of glucose (CMRGlc) initially reported by Fox and colleagues [42]. According to ANLSH, astrocytes take up glucose from the blood in a glutamate‐dependent manner and convert it into lactate, which is, in turn, released for further metabolization by neurons. Several studies suggest, however, that lactate function is much more than simply being an energetic substrate and that the shuttling of lactate is potentially bidirectional: in particular, in 2007, Simpson and colleagues argued that lactate transport was directed from neurons to astrocytes, leading to the hypothesis of the neuron‐to‐astrocytes lactate shuttle (NALSH) [43, 44]. Around this topic, a persistent scientific controversy has been generated [45, 46, 47, 48]. Lactate also acts as a modulator of synaptic plasticity [49]; recent studies suggested an involvement of lactate in cognitive and behavioral processes, including learning, memory [50, 51], and stress [52, 53] response, in addition to the signaling role that potentially influences vasodilation and the subsequent increase in CBF [54, 55].

The deep involvement of lactate in neuroenergetics was a key determinant of the first functional MRS studies, conducted with prolonged visual stimulation by Prichard and colleagues [4], Sappey‐Marinier and colleagues [56], and Frahm and colleagues [57]. All studies showed increases in lactate during the first minutes of stimulation of 57%, 150%, and 68%, respectively. Successively, several fMRS studies confirmed a significant increase in lactate following activations in the occipital lobe, frontal lobe, and cingulate cortex. Unlike studies focusing on glutamate (object of two meta‐analyses [32, 58] with 24 and 53 papers, respectively) and GABA (one meta‐analysis [32] with 22 papers), the number of fMRS publications focusing on lactate is relatively lower. This is mainly due to the greater difficulty in quantifying lactate, which in the healthy human brain has a concentration ranging between 0.5 and 1 mmol/L, much lower than the majority of metabolites. Furthermore, the doublet usually targeted for quantification is centered at 1.3 ppm and overlaps with unspecific signals assigned to macromolecules and lipids [59], making its quantification particularly challenging, especially at lower magnetic fields. Splitting the spectroscopy sequence into alternating stimulation and rest blocks reduces the number of transients available for averaging compared with a traditional ^1^H‐MRS acquisition. The associated difference procedure further decreases SNR and increases sensitivity to motion. fMRS optimization therefore requires careful selection of the sequence and its parameters, the stimulation paradigm, and the processing and quantification methods. These methodological choices vary widely across studies, contributing to substantial heterogeneity in the current fMRS literature.

This meta‐analysis aims to systematically evaluate the evidence for activity‐induced changes in brain lactate levels. Specifically, we aim to quantify the magnitude of lactate changes observed across different stimulation paradigms, magnetic field strengths, and brain regions and to assess the main methodological factors that may influence these results.

## 2 Meta‐Analysis

### 2.1 Search Strategy and Inclusion Criteria

Despite a recent consensus [60] for a standardization of proton MR spectroscopy, the heterogeneity in experimental design, spectroscopy sequences, and acquisition parameters contributes to the variability observed across studies. For example, an increase greater than 50% [4, 57, 61] of lactate concentration from baseline during visual stimulation has been reported, whereas in other studies, a smaller variation was found: Nichols and colleagues [62] found a not significant 2.5% increase in the anterior cingulate cortex (ACC), and Koush and colleagues [63] found a significant increase of 7.8% in the occipital lobe.

This meta‐analysis specifically focuses on fMRS studies of lactate in which a positive BOLD response is induced by block‐designed paradigms. The choice to focus on a positive BOLD response is associated with the current scarcity of fMRS studies on deactivation. Event‐related functional paradigms have been excluded from the current analysis due to the generally lower SNR and associated technical difficulties [64, 65]. On the other hand, whereas block‐designed stimuli allow the accumulation of many transients for averaging, long blocks result in poor temporal resolution of changes and potential habituation during the stimulus. To improve the stability of cognition during fMRS, the subject is often given attentional tasks (e.g., report with a button the change of shape or color of a fixation target).

The current meta‐analysis was conducted considering the papers published up to May 5, 2025. The following keys were used for four distinct searches on the PubMed database: “functional magnetic resonance spectroscopy,” “functional MRS,” “fMRS,” and “Magnetic resonance spectroscopy” (for this last one, some filters have been added, in particular “humans” and “clinical trial”). Criteria used for the papers, applied successively in this order, were as follows:

1. use functional ^1^H‐MRS in in vivo human brain
2. no invasive stimulations or drugs
3. application on healthy humans or having a control group if in a disease population
4. paper written in English and published in a peer‐reviewed journal
5. investigation of lactate concentration change during prolonged (> 30 s) stimuli inducing a positive BOLD response
6. presence of the needed statistical information, including mean and standard deviation (or SEM)

**FIGURE 1:**
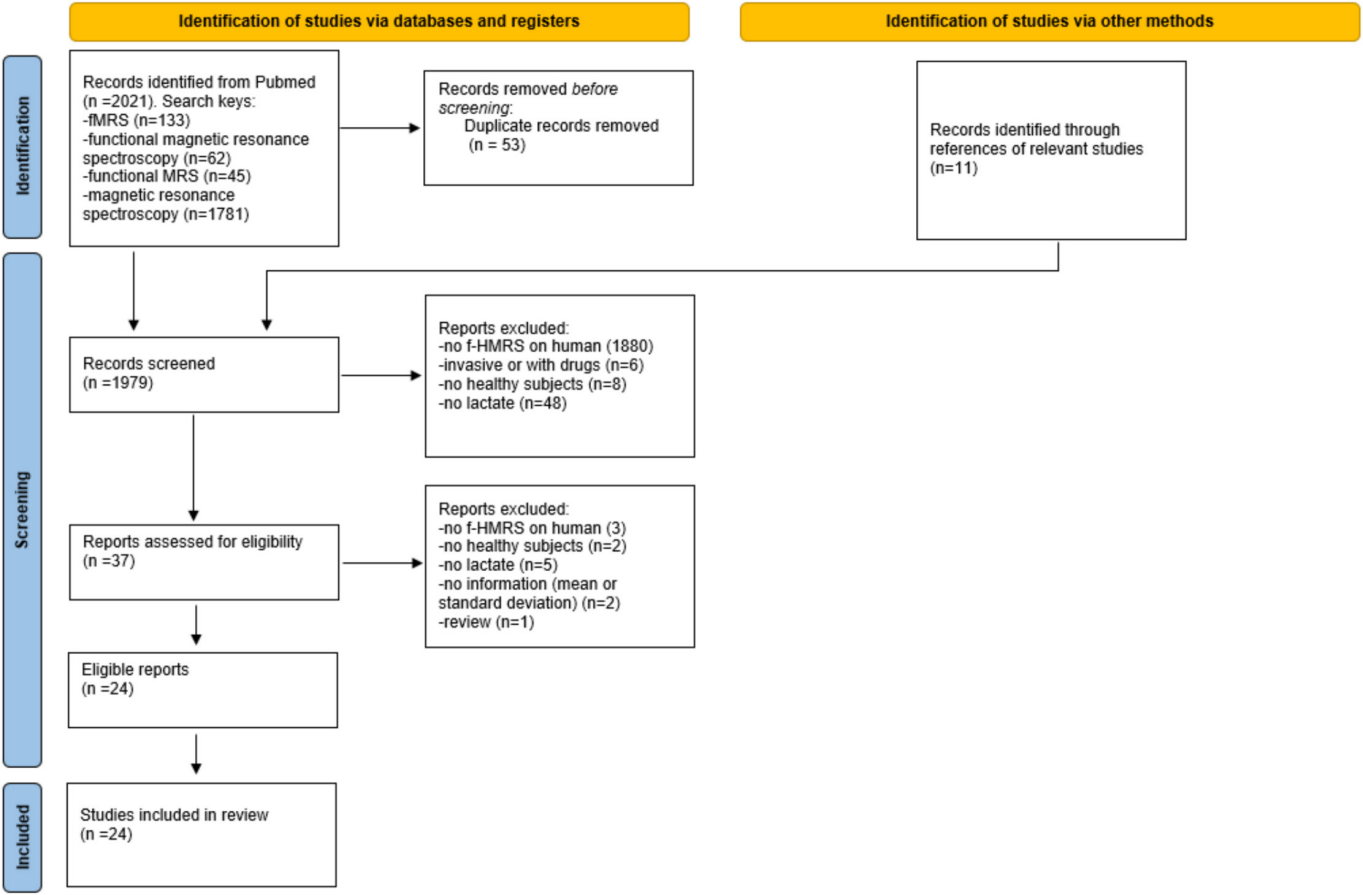
PRISMA flowchart reporting study identification, screening, eligibility, and subsequent inclusion process.

The identification, screening, and inclusion process are reported in Figure 1 with the PRISMA flowchart [66]. The initial database search yielded a total of 2032 articles, distributed across search terms as follows: 62 for “functional magnetic resonance spectroscopy,” 45 for “functional MRS,” 133 for “fMRS,” and 1781 for “Magnetic resonance spectroscopy” (with filters for “humans” and “clinical trial”). An additional 11 articles were identified through references of relevant studies [67]. After removing duplicates, the papers underwent a first screening based on title and abstract only. Thirty‐seven papers were retained after this step; in particular, most papers were rejected for being off‐topic. The remaining papers were then assessed for eligibility through a full‐text review. Twenty‐four papers were finally retained for the meta‐analysis. In particular, the article by Sappey‐Marinier and colleagues [56], reporting an increase of lactate of 150%, was generally in line with our criteria, but, due to the absence of standard deviation values, it was discarded from the meta‐analysis. The “aged” condition (participants over 60 years) from the paper of Urrila and colleagues [68] was excluded, as its age range was not comparable to that of the other included studies.

The meta‐analysis was conducted by grouping papers according to the following characteristics:

1. static magnetic field B_0_ (high: 1.5, 2, 2.1, 3, and 4 T; ultra‐high: 7 and 9.4 T)
2. echo time (short TE: 5–30 ms; long TE: 144–288 ms)
3. brain region of voxel of interest (VOI) placement: occipital lobe, motor cortex, ACC, and inferior frontal gyrus (IFG)

For each study, the spectroscopic sequence (STEAM [69], PRESS [70], semiLASER [71], and SPECIAL [72]), the stimulus type, the sample size, and the VOI dimensions were additionally extracted.

### 2.2 Results

Table 1 shows the 24 papers finally included in the current meta‐analysis [4, 5, 13, 34, 57, 61, 62, 63, 68, 73, 74, 75, 76, 77, 78, 79, 80, 81, 82, 83, 84, 85, 86, 87], listed based on the year of publication, along with the relevant study details. The reported information includes stimulus type, brain region of interest, static magnetic field strength, sequence, repetition time (TR) and TE, number of subjects (*N*), VOI size in cubic centimeters, stimulus duration, and whether lactate changes were reported as a single value measure or as a time‐resolved measure. In the case of a time‐resolved measure, the values considered in this analysis correspond either to those reported in the abstract or to those deemed by the authors as the most representative. The 95% confidence intervals (CIs) were calculated using the standard deviations reported in the respective papers. If not explicitly provided, CIs were estimated from the reported *p*‐values or derived through standard uncertainty propagation formulas. The CI estimation from *p*‐value was done for four studies [68, 74, 77, 80], whereas the error propagation was done for two studies [57, 62]. Table 1 shows that the number of fMRS studies reporting lactate changes has strongly increased in recent years, possibly due to the adoption of more advanced spectroscopic sequences such as semiLASER and SPECIAL and the use of ultra‐high magnetic field strengths. All studies were conducted on groups of healthy subjects within a specific age range, usually between 18 and 35 years. The mean age across all papers was 26.9 ± 5.4 years (mean ± SD), thus introducing minimal age‐related variability in the current meta‐analysis. Even though Kim and colleagues [77], Nichols and colleagues [62], and Urrila and colleagues [68, 74] focused their studies on female groups, the cumulative percentage of females in the various studies was 51% ± 29% (mean ± SD). To assess the variability of the various protocols used, we evaluated the statistical parameter I^2^, which represents the percentage of variability across studies that arises from true differences rather than sampling error. The heterogeneity across studies included in this meta‐analysis is high (I^2^ = 74%), which is expected due to the high variability of experimental procedures. Methodological differences likely contribute to the wide range of observed lactate changes; however, I^2^ values are high also for studies using high magnetic field strength (I^2^ = 83%), short TE (I^2^ = 74%), and visual areas (I^2^ = 82%). I^2^ = 0% was obtained for studies using ultra‐high magnetic field strength, which could reflect the low heterogeneity but may be affected by a lower number of studies compared to the previous cases. The I^2^ statistic was not computed for these other subdivisions (long‐TE, motor area, ACC area, and IFG area) owing to the small number of included studies, which would render the estimate unreliable. These values are roughly in line with the I^2^ values reported by Pasanta and colleagues in a meta‐analysis on GABA and glutamate [32], ranging between 60% and 95%.

**TABLE 1:** Lactate fMRS studies included in the meta‐analysis. For each of them, stimulus, brain region, B_0_, spectroscopic sequence, number of subjects (*N*), VOI size, experiment stimulus duration, and output variation are reported.

Author	Year	Stimulus	Brain region	B_0_ (T)	Sequence/TR (s)/TE (ms)	*N*	VOI (cm^3^)	Exp design (min)	Single value measure	Time‐resolved measure
Prichard et al. [4]	1991	Visual	Visual cortex	2.1	PRESS	5	—	> 5	No	Yes
Kuwabara et al. [73]	1995	Exercise	Motor cortex	1.5	PRESS/1.5/270	7	56	> 5	Yes	No
Frahm et al. [57]	1996	Visual	Visual cortex	2	STEAM/6/20	11	11	> 5	No	Yes
Urrila et al. [74]	2003	Cognitive	IFG	1.5	PRESS/1.5/288	12	6.9	< 5	No	Yes
Urrila et al. [68]	2004	Cognitive	IFG	1.5	PRESS/1.5/288	13	6.6	> 5	Yes	No
Mangia et al. [5]	2007a	Visual	Visual cortex	7	STEAM/5/6	12	8.8	> 5	Yes	No
Mangia et al. [75]	2007b	Visual	Visual cortex	7	STEAM/5/6	12	8.8	< 5	No	Yes
Lin et al. [61]	2010a	Visual	Visual cortex	3	PRESS/2/30	12	16	< 5	Yes	No
Lin et al. [61]	2010b	Visual	Visual cortex	3	PRESS/2/30	12	16	< 5	Yes	No
Lin et al. [61]	2010c	Visual	Visual cortex	3	PRESS/2/30	12	16	< 5	Yes	No
Lin et al. [76]	2012	Visual	Visual cortex	7	STEAM/3/15	10	8	> 5	Yes	No
Kim et al. [77]	2013	Visual	ACC	3	PRESS/2/30	23	7.2	> 5	Yes	No
Schaller et al. [78]	2013	Visual	Visual cortex	7	SPECIAL/5/6	10	8.8	5	Yes	No
Schaller et al. [13]	2014	Exercise	Motor cortex	7	SPECIAL/7.5/12	11	5.8	5	Yes	No
Bednařík et al. [79]	2015	Visual	Visual cortex	7	semiLASER/5/26	12	8	> 5	Yes	No
Mekle et al. [80]	2017	Visual	Visual cortex	7	SPECIAL/5/6	21	8	> 5	Yes	No
Bednařík et al. [81]	2018a	Visual	Visual cortex	7	semiLASER/5/26	12	8	> 5	Yes	No
Bednařík et al. [81]	2018b	Visual	Visual cortex	7	semiLASER/5/26	12	8	> 5	Yes	No
Koush et al. [82]	2019	Exercise	Motor cortex	4	semiLASER/3.3/144	10	13	> 5	Yes	No
Boillat et al. [34]	2020	Visual	Visual cortex	7	SPECIAL/7.5/16	20	5.8	5	Yes	No
Fernandes et al. [83]	2020	Visual	Visual cortex	7	semiLASER/5/144	6	12	> 5	No	Yes
van Vugt et al. [84]	2020	Exercise/learning	Motor cortex	3	PRESS/2.5/19	25	18	> 5	No	Yes
Koush et al. [63]	2021	Visual	Visual cortex	4	STEAM/2.7/20	21	13	< 5	Yes	No
DiNuzzo et al. [85]	2022	Visual	Visual cortex	7	STEAM/3/7	16	10	< 5	Yes	No
Dorst et al. [86]	2022	Visual	Visual cortex	9.4	semiLASER/5/24	10	5.4	> 5	Yes	No
Nichols et al. [62]	2023a	Pain	ACC	7	STEAM/10/5	17	15	< 5	Yes	No
Nichols et al. [62]	2023b	Pain	ACC	7	STEAM/10/5	17	15	< 5	Yes	No
Morelli et al. [87]	2025	Exercise	Motor cortex	3	semiLASER/2/28	23	16	> 5	No	Yes

**FIGURE 2:**
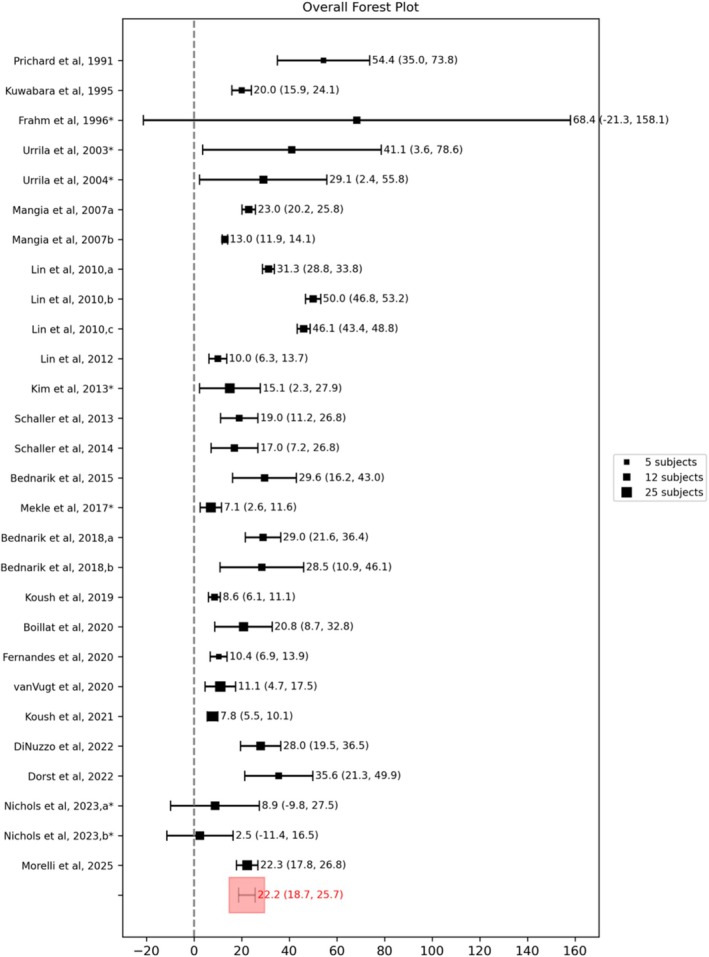
Overall forest plot of the concentration change of lactate. Squares represent the mean and their size is proportional to the number of subjects. For each data point, the 95% CI is reported. Asterisks indicate studies for which standard deviation values were estimated from reported *p*‐values or derived through error propagation methods.

Figure 2 shows the forest plot of the percentage change of lactate concentration following stimulation for all the studies considered. Although the number of included papers is 24, in the forest plot, 28 data points are reported because some papers included more than one experimental condition. In particular, in Lin et al. (2010) [61], the visual stimulation consisted of a black‐and‐white radial checkerboard reversing its contrast at frequencies of 4, 8, and 16 Hz; therefore, three different results are reported here as Lin et al. (2010a, 2010b, 2010c). Similarly, Bednařík et al. (2018a, 2018b) [81] used two different visual stimuli (chromatic and achromatic), both simultaneously rotated and expanded/contracted at the same frequency of 2 Hz. In Nichols et al. (2023), the division into two groups was made due to the different levels of pain intensity (low and moderate) included in the study. On the other hand, Mangia et al. (2007a) [5] and Mangia et al. (2007b) [75] represent two different papers. Because all the considered papers used a block design for stimulation, the percentage change in concentration is taken as the percentage difference between the stimulation (STIM) and the rest (REST) epochs. An overall mean lactate concentration increase of 22.2% was found (18.7%–25.7% with a 95% CI, I^2^ = 74%) and was found to be statistically significant (one‐sample two‐tailed *t*‐test, *p* < 0.001). The large CI reported in some studies [4, 34, 57, 81] indicates also a strong subject‐related variability, which can be due to the low reliability of lactate quantification because of its low concentration in vivo in healthy brains and especially to the presence of macromolecules and lipids in the same resonance range.

#### 2.2.1 Effect of Echo Time and B_0_

**FIGURE 3:**
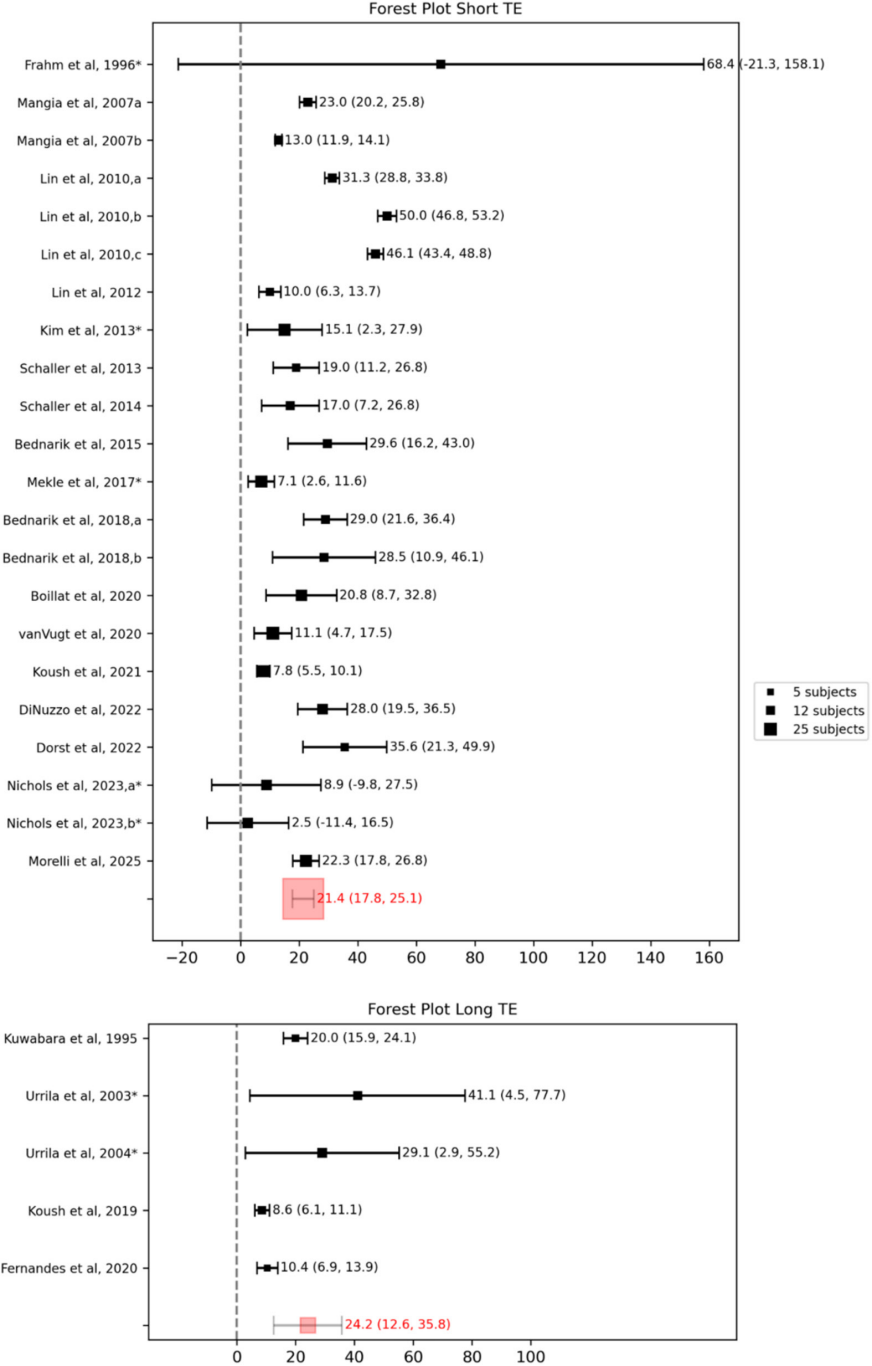
Forest plots of studies classified according to TE. Asterisks indicate studies for which standard deviation values were estimated from reported *p*‐values or derived through error propagation methods.

The TE significantly affects spectral features and processing. A short‐TE sequence has a higher SNR compared to a long‐TE sequence but can result in major baseline irregularities. The forest plot in Figure 3 shows the difference in lactate changes using short‐TE and long‐TE sequences; most studies prioritized SNR by choosing shorter TEs, with a minimum value of 5 ms [62] using the STEAM sequence. Only five papers used long‐TE sequences, respectively, 144 ms [82, 83] with a semiLASER sequence, 270 ms [73] with a PRESS sequence, and 288 ms [68, 74] with a PRESS sequence. Furthermore, these papers have a smaller sample size with respect to the average of 15 of the short‐TE ones (6 subjects for Fernandes et al. 2020 at 7 T, 7 for Kuwabara et al. 1995 at 1.5 T, 10 for Koush et al. 2019 at 4 T, 12 for Urrila et al. 2003, and 13 for Urrila et al. 2004 both at 1.5 T). No statistically significant variations were found between the two TE groups. TE affects the T_2_‐weighting of metabolites, usually not accounted for during quantification. Metabolite T_2_ is influenced by the strength of the magnetic field used [88] but, more importantly, by the environment. For instance, Wyss and colleagues [89] demonstrated at 3 T that lactate exhibits different T_2_ values across brain regions, ranging from 99 ms in the occipital lobe to 159 ms in the periventricular white matter. Similarly, Jouvensal and colleagues [90] found in rats at 3 T that the T_2_ of intracellular lactate is significantly shorter (33 ms) compared to the extracellular lactate (230 ms), a difference attributed to the higher viscosity and lower molecular mobility inside cells versus the extracellular space. As a consequence, long TE is likely associated with heavier weighting of the extracellular compartment. The overall T_2_ of the lactate pool (intracellular + extracellular) remains considerably longer than that of macromolecules [91], which, due to their larger size and rigidity, exhibit limited molecular motion and thus much shorter T_2_ times. In long‐TE spectroscopy sequences, signals from metabolites with short T_2_ are strongly attenuated, which reduces or eliminates the interfering signal from macromolecules in the lactate spectral range. As a result, the tendency to increased lactate change observed in long TE compared to short TE studies (24.2% with 95% CI [12.3%–36.0%] for long TE; 21.4% with 95% CI [17.8%–25.1%] for short TE) may reflect a bias toward the extracellular lactate and thus an increased weighting of intracellular transport versus intracellular metabolization.

**FIGURE 4:**
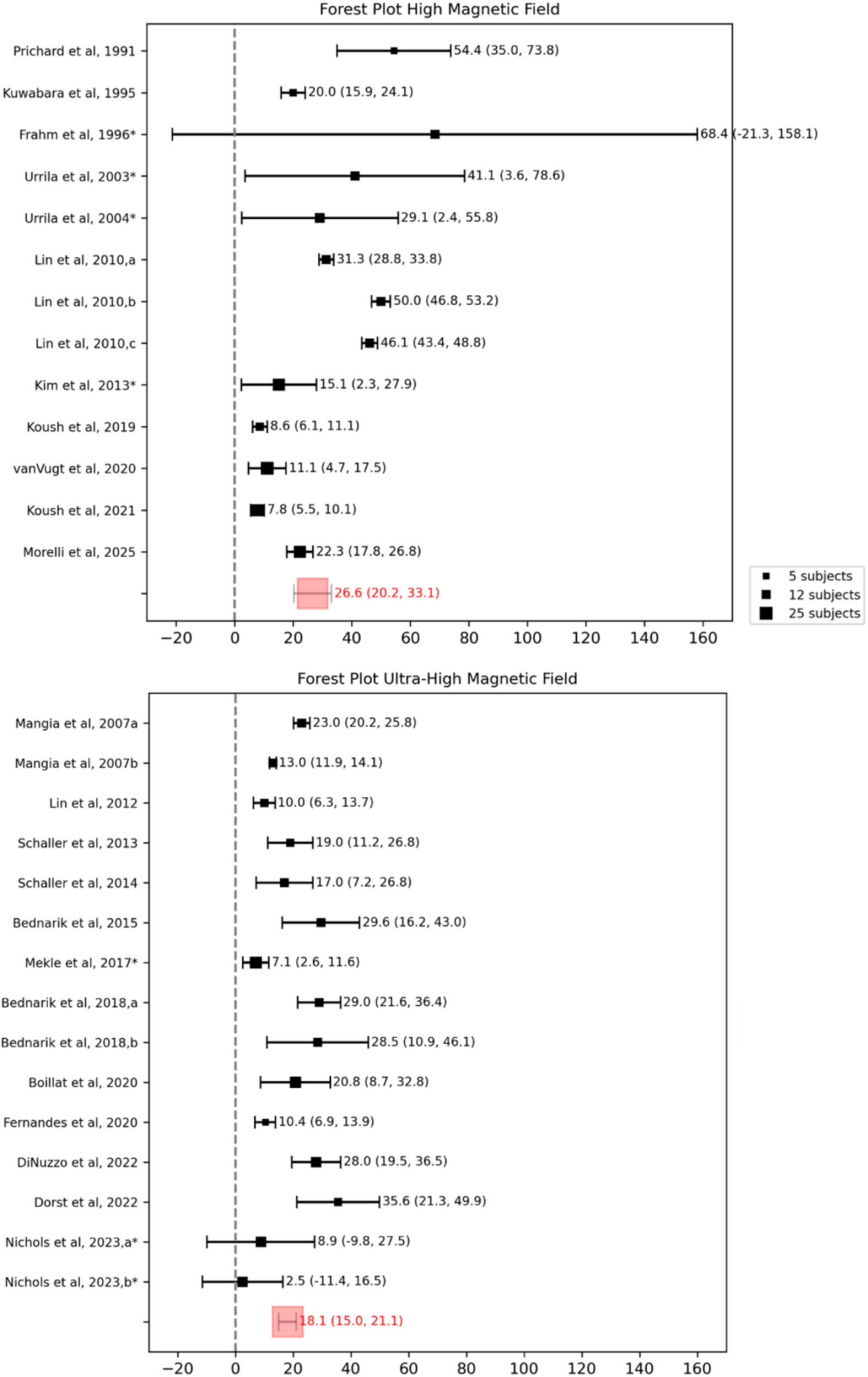
Forest plots of studies classified according to magnetic field. Asterisks indicate studies for which standard deviation values were estimated from reported *p*‐values or derived through error propagation methods.

The magnetic field used for the experiment has a strong impact on the quantification reliability of lactate [67]. Figure 4 shows the forest plots obtained by classifying the studies according to magnetic field strength: high (from 1.5 to 4 T) or ultra‐high (7 and 9.4 T). In particular, the most used field is 7 T (12 papers), followed by 3 T (4 papers). Higher magnetic fields allow for better separation of the peaks of the various metabolites and have higher sensitivity (higher SNR) than lower fields but have the drawback of greater susceptibility to artifacts. The Cramér–Rao lower bounds (CRLB), a value related to the fit error of each single metabolite and often used for data quality [60], benefit from the use of higher field strengths. In particular, Paiva and colleagues [92] and Terpstra and colleagues [93] showed that in healthy volunteers, the mean lactate CRLB decreases significantly with increasing magnetic field (from 1.5 to 3 T and from 3 to 7 T, respectively), whereas Deelchand and colleagues [94] confirmed, with simulations, the decrease of CRLB at magnetic fields ranging between 1.5 and 11.7 T. Typically, for lactate, the CRLB threshold is set at 35%; eight papers at ultra‐high magnetic fields reported the value of the CRLB at an overall 10.2% ± 3.4% (mean ± SD). Figure 3 shows that studies conducted at higher magnetic fields tend to have a decreased percentage change in lactate concentration compared to lower fields (18.1% with 95% CI [15.0%–21.1%] for ultra‐high magnetic fields; 26.6% with 95% CI [20.2%–33.1%] for high magnetic fields), but no significant variation was found between the two groups.

An advantage of ultra‐high magnetic fields is the possibility to decrease the VOI dimension, maintaining a high SNR, thus reducing contamination from adjacent tissues. Contamination can cause artifacts and strongly influence the observed metabolic change via inclusion of tissues where a metabolic response to stimulation is expected to be negligible or absent (nonactivated gray matter, white matter, and cerebrospinal fluid). The degree of partial volume averaging may be estimated by the relative volume of voxels exhibiting a positive BOLD response within the spectroscopic VOI. Only a few papers (*n* = 7) report the BOLD change within the VOI, which is 2.1% ± 0.7% on average, and a few others (*n* = 3) report the percentage of active voxels within the VOI, which is 34% ± 12% on average. In general terms, a larger VOI was used in earlier, low field studies, for example, Kuwabara et al. [73] at 1.5 T used a 56‐cm^3^ VOI, whereas Dorst et al. [86] at 9.4 T used a 5.4‐cm^3^ one; furthermore, the greatest CI reported in this meta‐analysis with Frahm et al. [57, 74] can be associated with the small size of its VOI (11 cm^3^) along with the low B_0_ of 2 T.

#### 2.2.2 Effect of Brain Region and Stimulus Type

**FIGURE 5:**
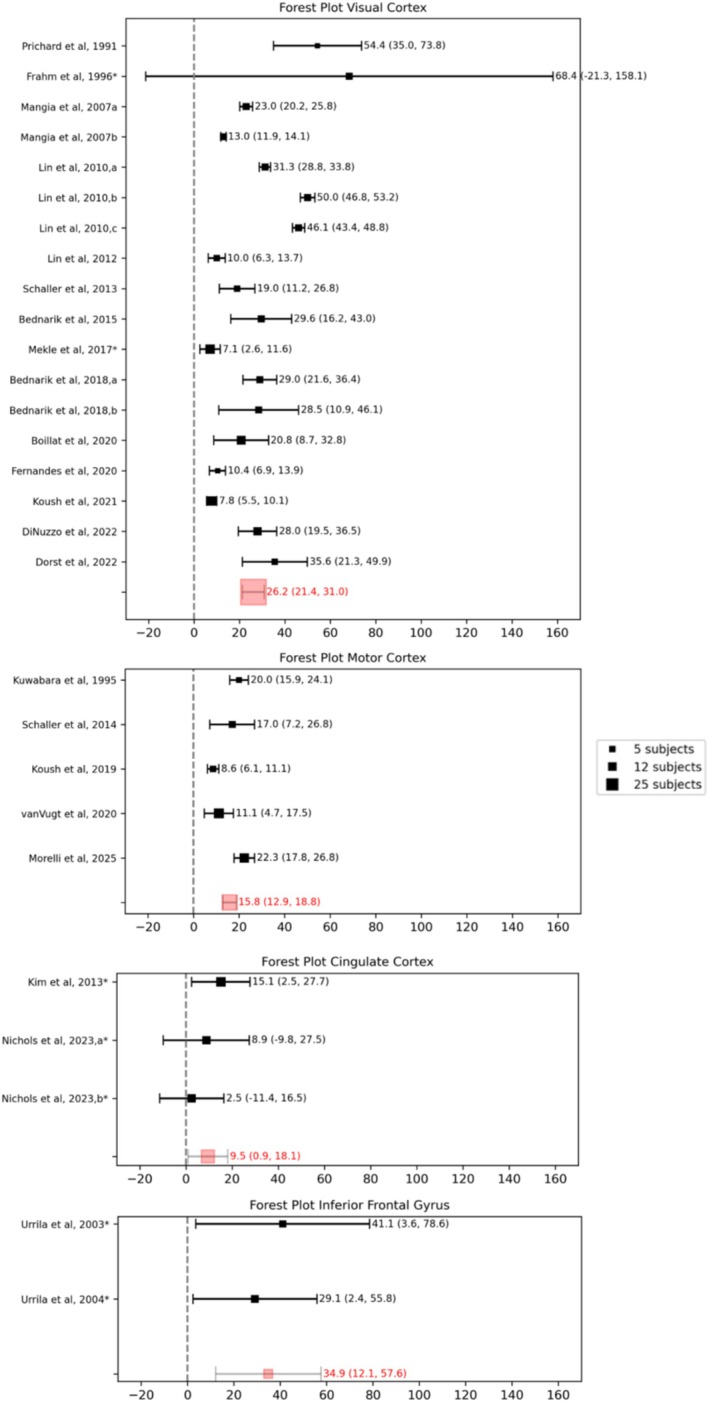
Forest plots of studies classified according to investigated brain region. Asterisks indicate studies for which standard deviation values were estimated from reported *p*‐values or derived through error propagation methods.

The changes in lactate concentration in different brain regions are reported in Figure 5. The brain region is obviously associated with the stimulus used to activate the area. The most commonly studied brain area is the visual cortex, with the VOI usually including the primary visual area V1. The choice of the occipital lobe and visual stimuli is probably related to the easiest induction of stable activation and to the very simple design of the stimulus, which is usually a full‐radial flickering checkerboard. The range of flickering frequencies used was between 2 Hz [81] and 16 Hz [61]. In particular, Bednařík et al. demonstrated that there is no statistically significant difference between the change in metabolism under chromatic (Bednařík et al. 2018a) and achromatic (Bednařík et al. 2018b) stimulation, flickering at the same frequency, even though those stimulations are assumed to activate two different neuronal populations (blob and interblob) [95, 96]. Instead, Lin and colleagues [61] used three different flickering frequencies (4, 8, and 16 Hz) and showed a stronger coupling between CBF and CMRO2 with lower frequency, in line with Vafaee and Gjedde [97] and Lin and colleagues [98]. DiNuzzo and colleagues [85] showed that using an unperceived flickering stimulus (30 Hz), the BOLD response is still maintained, but no changes in lactate are found, whereas lower frequency stimulation (7.5 Hz) elicits a robust lactate increase in V1. Four papers studied lactate in the motor cortex, either with purely motor tasks like finger tapping [13, 73, 82] or calibrated fist clenching [87], whereas van Vugt and colleagues [84] used an audio‐motor learning task. Cingulate cortex VOI placement was used with painful stimuli by Nichols and colleagues [62] or visual cognitive stimuli by Kim et al. [77]. Finally, fMRS analysis conducted in the IFG by Urrila et al. [68, 74] used a cognitive task based on a verbal test of silent word generation. The higher value of lactate change reported with visual tasks can be related to the generally higher SNR in the occipital lobe. Statistical differences in lactate change were found when considering studies with VOI localized in the occipital lobe (mean 26.2% with 95% CI [21.4%–31.0%]) compared to motor areas (mean 15.8% with 95% CI [12.9%–18.8%]) and ACC (mean 9.5% with 95% CI [0.9%–18.1%]) (two‐tailed *t*‐test with *p* < 0.05 and *p* < 0.01, respectively). No statistical differences were found with all the other comparisons.

Rest and stimulation epoch duration during the spectroscopy acquisition is usually in the range of 4 and 6 min, related also to the TR and consequently to the number of transients averaged in a block that can vary from 40 scans [34] to 64 scans [79].

The duration of stimulation may influence lactate dynamics, primarily through physiological adaptation phenomena. Indeed, during prolonged stimulation, tissues frequently exhibit metabolic habituation or enhanced efficiency in lactate clearance mechanisms [99]. However, the BOLD signal typically exhibits an initial transient overshoot followed by a prolonged, stable plateau [100]. Similarly, local field potentials (LFPs), reflecting pooled synaptic activity, demonstrate rapid habituation, settling into a sustained steady state during continuous stimulation [101]. Figure S1 presents a scatter plot illustrating the percentage change in lactate relative to the duration of the stimulation blocks across the included studies. Notably, no clear trend is observed, and the correlation between these variables is not statistically significant (*R* = 0.33, *p* = 0.09). This indicates that the duration of the stimulation blocks does not significantly influence the magnitude of lactate changes across studies, suggesting that an overall metabolic steady state is achieved during prolonged stimulation regardless of how long the stimulation block lasts, within the range included in this meta‐analysis. It should be noted, however, that the few time‐resolved studies available report a dynamic pattern in lactate concentration, with the peak concentration reached during the early phase of stimulation, followed by a gradual decrease for the remainder of the stimulation period, suggesting that initial metabolic responses may not conform to a simple steady‐state model [56, 75]. Further investigation is needed to address this issue.

## 3 Discussion and Conclusions

Despite the considerable heterogeneity resulting from the different protocols applied, all the studies included in this meta‐analysis consistently demonstrate that intense brain activity and the resulting positive BOLD signal are associated with increased lactate concentrations. Probably due to an overall high heterogeneity, even within studies classified according to TE, magnetic field, and brain area, we found only a single statistically significant difference in lactate response between the various classifications, namely, between studies conducted in visual areas and motor areas (*p* < 0.05) and in visual areas and ACC (*p* < 0.01). This difference likely reflects an overall different amplitude of neurometabolic response to different classes of stimuli. Notably, the number of studies in some classes is low (e.g., we found only 5 long TE studies compared to the 19 in short TE studies), reducing the robustness of conclusions regarding these classes.

The correlation between BOLD and Lac changes indicates an association between increased energy demand and functional response (neurometabolic coupling); its relationship with an increase in oxidative metabolism is debated and generally based on consideration of simultaneous change of other metabolites (rise in glutamate and decrease of aspartate [81]). The lactate increase is linked to a corresponding rise in Pyr concentration (due to their dynamic equilibrium) and thus to the activation of Pyr dehydrogenase. According to Boillat and colleagues [34], the positive variation in Glu and Lac during stimulation can be seen as a robust marker of neuronal activation in the human visual cortex. However, DiNuzzo and colleagues [85] found that although the evoked BOLD signal does not change using a checkerboard flickering at perceived (15 Hz) or nonperceived (30 Hz) frequencies, the variation in Lac with respect to baseline is significant only in the first case. This uncoupling between BOLD and metabolic response suggests that the physiological mechanisms underlying BOLD and energy metabolism do not overlap in every condition and that, in general, neurometabolic and neurovascular coupling reflect physiological phenomena that are at least partially regulated through different mechanisms. This meta‐analysis indicates an average 22% increase in lactate concentration during stimulation. Taking also into account the work by Boillat and colleagues [34], reporting a decrease in lactate and glutamate in deactivated areas, task‐related changes in lactate emphasize lactate's key role in brain function.

This meta‐analysis also reveals substantial methodological heterogeneity across studies. This variability extends to processing and quantification procedures, which were outside the scope of the present work. A source of variability might also be related to the handling of linewidth changes associated with the BOLD effect, which can lead to overestimation of metabolite concentrations [102, 103]. Not all studies included in the meta‐analysis reported the use of line matching for linewidth adjustment, and in many cases (especially in older studies), the processing and quantification approaches were not described in sufficient detail to enable explicit comparisons or to identify specific sources of heterogeneity. Consequently, this methodological variability may have reduced the comparability of lactate estimates across studies. It should also be noted that, due to the limited number of available studies, we did not assess interactions between methodological variables that are inherently interrelated, such as magnetic field strength, TE, and brain region. For example, only one long‐TE study was conducted at an ultra‐high magnetic field. The limitation posed by the small number of studies is particularly evident for the subgroup with VOIs located in the IFG. Specifically, only two studies [68, 74], both conducted by the same research group, have focused on this region, making it difficult to draw broader conclusions regarding its involvement.

Finally, the MRS studies included in this meta‐analysis were generally designed to highlight metabolic dynamics in response to stimulation, meaning that publication bias could potentially affect the results. To address this, we visualized the included studies using a funnel plot (Figure S2), plotting study effect sizes as a function of their standard errors (SEs). Each study is color‐coded by publication year. Although some older studies exhibit larger SEs, this pattern is not strictly consistent, as several older studies also appear in the upper portion of the plot. Conversely, more recent studies tend to cluster near the top of the funnel, reflecting lower result dispersion—likely driven by larger sample sizes and methodological improvements.

To formally quantify the visual assessment of the funnel plot, we conducted an Egger's regression test for asymmetry. The test yielded a nonsignificant result (*p* = 0.34), thus indicating no evidence of publication bias.

Future research would benefit from adopting more standardized experimental procedures to improve the homogeneity, reproducibility, and interpretability of results, an essential step toward extending the use of lactate fMRS to clinical populations.

## Author Contributions

**Luca Cairone:** conceptualization, data curation, formal analysis, writing – original draft, writing – review and editing. **Maria Guidi:** conceptualization, writing – review and editing. **Matteo Mancini:** conceptualization, writing – review and editing. **Federico Giove:** conceptualization, writing – review and editing, supervision.

## Conflicts of Interest

The authors declare no conflicts of interest.

## Supporting information


**Figure S1:** Funnel plot of the studies included in the meta‐analysis. Each study is color‐coded by year of publication.
**Figure S2:** Scatter plot with effect size and block stimulation duration.

## Data Availability

The data supporting these meta‐analysis findings are available within the article. All relevant data have been included. Any additional materials generated or analyzed during this work, such as tables and statistical analyses, are available from the corresponding author upon reasonable request.

## References

[1] C. I. Mark , E. L. Mazerolle , and J. J. Chen , “Metabolic and Vascular Origins of the BOLD Effect: Implications for Imaging Pathology and Resting‐State Brain Function,” Journal of Magnetic Resonance Imaging 42, no. 2 (2015): 231–246, 10.1002/jmri.24786. 25727523

[2] I. B. Ip , A. Berrington , A. T. Hess , A. J. Parker , U. E. Emir , and H. Bridge , “Combined fMRI‐MRS Acquires Simultaneous Glutamate and BOLD‐fMRI Signals in the Human Brain,” NeuroImage 155 (2017): 113–119, 10.1016/j.neuroimage.2017.04.030. 28433623 PMC5519502

[3] S. Mangia , F. Giove , I. Tkáč , et al., “Metabolic and Hemodynamic Events After Changes in Neuronal Activity: Current Hypotheses, Theoretical Predictions and In Vivo NMR Experimental Findings,” Journal of Cerebral Blood Flow & Metabolism 29, no. 3 (2008): 441–463, 10.1038/jcbfm.2008.134. 19002199 PMC2743443

[4] J. Prichard , D. Rothman , E. Novotny , et al., “Lactate Rise Detected by 1H NMR in Human Visual Cortex During Physiologic Stimulation,” Proceedings of the National Academy of Sciences of the United States of America 88, no. 13 (1991): 5829–5831, 10.1073/pnas.88.13.5829. 2062861 PMC51971

[5] S. Mangia , I. Tkác , R. Gruetter , P. F. Van de Moortele , B. Maraviglia , and K. Uğurbil , “Sustained Neuronal Activation Raises Oxidative Metabolism to a New Steady‐State Level: Evidence From ^1^H NMR Spectroscopy in the Human Visual Cortex,” Journal of Cerebral Blood Flow and Metabolism: Official Journal of the International Society of Cerebral Blood Flow and Metabolism 27, no. 5 (2007): 1055–1063, 10.1038/sj.jcbfm.9600401. 17033694

[6] G. E. Dwyer , A. R. Craven , J. Bereśniewicz , et al., “Simultaneous Measurement of the BOLD Effect and Metabolic Changes in Response to Visual Stimulation Using the MEGA‐PRESS Sequence at 3 T,” Frontiers in Human Neuroscience 15 (2021): 644079, 10.3389/fnhum.2021.644079. 33841118 PMC8024522

[7] P. G. Mullins , L. M. Rowland , R. E. Jung , and W. L. Sibbitt, Jr. , “A Novel Technique to Study the Brain's Response to Pain: Proton Magnetic Resonance Spectroscopy,” NeuroImage 26, no. 2 (2005): 642–646, 10.1016/j.neuroimage.2005.02.001. 15907322

[8] J. Archibald , E. L. MacMillan , C. Graf , P. Kozlowski , C. Laule , and J. L. K. Kramer , “Metabolite Activity in the Anterior Cingulate Cortex During a Painful Stimulus Using Functional MRS,” Scientific Reports 10, no. 1 (2020): 19218, 10.1038/s41598-020-76263-3. 33154474 PMC7645766

[9] A. Gussew , R. Rzanny , M. Erdtel , et al., “Time‐Resolved Functional ^1^H MR Spectroscopic Detection of Glutamate Concentration Changes in the Brain During Acute Heat Pain Stimulation,” NeuroImage 49, no. 2 (2010): 1895–1902, 10.1016/j.neuroimage.2009.09.007. 19761852

[10] A. Floyer‐Lea , M. Wylezinska , T. Kincses , and P. M. Matthews , “Rapid Modulation of GABA Concentration in Human Sensorimotor Cortex During Motor Learning,” Journal of Neurophysiology 95, no. 3 (2006): 1639–1644, 10.1152/jn.00346.2005. 16221751

[11] D. Apšvalka , A. Gadie , M. Clemence , and P. G. Mullins , “Event‐Related Dynamics of Glutamate and BOLD Effects Measured Using Functional Magnetic Resonance Spectroscopy (fMRS) at 3 T in a Repetition Suppression Paradigm,” NeuroImage 118 (2015): 292–300, 10.1016/j.neuroimage.2015.06.015. 26072254

[12] V. Bezalel , R. Paz , and A. Tal , “Inhibitory and Excitatory Mechanisms in the Human Cingulate‐Cortex Support Reinforcement Learning: A Functional Proton Magnetic Resonance Spectroscopy Study,” NeuroImage 184 (2019): 25–35, 10.1016/j.neuroimage.2018.09.016. 30201464

[13] B. Schaller , L. Xin , K. O'Brien , A. W. Magill , and R. Gruetter , “Are Glutamate and Lactate Increases Ubiquitous to Physiological Activation? A ^1^H Functional MR Spectroscopy Study During Motor Activation in Human Brain at 7 Tesla,” NeuroImage 93, no. Pt 1 (2014): 138–145, 10.1016/j.neuroimage.2014.02.016. 24555953

[14] C. Chen , H. P. Sigurdsson , S. E. Pépés , et al., “Activation Induced Changes in GABA: Functional MRS at 7 T With MEGA‐sLASER,” NeuroImage 156 (2017): 207–213, 10.1016/j.neuroimage.2017.05.044. 28533117

[15] O. Volovyk and A. Tal , “Increased Glutamate Concentrations During Prolonged Motor Activation as Measured Using Functional Magnetic Resonance Spectroscopy at 3T,” NeuroImage 223 (2020): 117338, 10.1016/j.neuroimage.2020.117338. 32896636

[16] D. A. Butterfield and B. Halliwell , “Oxidative Stress, Dysfunctional Glucose Metabolism and Alzheimer Disease,” Nature Reviews. Neuroscience 20, no. 3 (2019): 148–160, 10.1038/s41583-019-0132-6. 30737462 PMC9382875

[17] N. G. Rummel and D. A. Butterfield , “Altered Metabolism in Alzheimer Disease Brain: Role of Oxidative Stress,” Antioxidants & Redox Signaling 36, no. 16–18 (2022): 1289–1305, 10.1089/ars.2021.0177. 34416829 PMC9229240

[18] S. Prabakaran , J. E. Swatton , M. M. Ryan , et al., “Mitochondrial Dysfunction in Schizophrenia: Evidence for Compromised Brain Metabolism and Oxidative Stress,” Molecular Psychiatry 9, no. 7 (2004): 684–697, 10.1038/sj.mp.4001511. 15098003

[19] P. Khaitovich , H. E. Lockstone , M. T. Wayland , et al., “Metabolic Changes in Schizophrenia and Human Brain Evolution,” Genome Biology 9, no. 8 (2008): R124, 10.1186/gb-2008-9-8-r124. 18681948 PMC2575514

[20] D. Boison and C. Steinhäuser , “Epilepsy and Astrocyte Energy Metabolism,” Glia 66, no. 6 (2017): 1235–1243, 10.1002/glia.23247. 29044647 PMC5903956

[21] J. M. Rho and D. Boison , “The Metabolic Basis of Epilepsy,” Nature Reviews. Neurology 18, no. 6 (2022): 333–347, 10.1038/s41582-022-00651-8. 35361967 PMC10259193

[22] R. Taylor , B. Schaefer , M. Densmore , et al., “Increased Glutamate Levels Observed Upon Functional Activation in the Anterior Cingulate Cortex Using the Stroop Task and Functional Spectroscopy,” Neuroreport 26, no. 3 (2015): 107–112, 10.1097/wnr.0000000000000309. 25536234 PMC4323558

[23] J. Chiappelli , Q. Shi , S. A. Wijtenburg , et al., “Glutamatergic Response to Heat Pain Stress in Schizophrenia,” Schizophrenia Bulletin 44, no. 4 (2018): 886–895, 10.1093/schbul/sbx133. 29036718 PMC6007227

[24] L. A. Jelen , S. King , C. M. Horne , D. J. Lythgoe , A. H. Young , and J. M. Stone , “Functional Magnetic Resonance Spectroscopy in Patients With Schizophrenia and Bipolar Affective Disorder: Glutamate Dynamics in the Anterior Cingulate Cortex During a Working Memory Task,” European Neuropsychopharmacology 29, no. 2 (2019): 222–234, 10.1016/j.euroneuro.2018.12.005. 30558824

[25] A. Ophey , E. Farrher , N. Pagel , et al., “Visuo‐Spatial Processing Is Linked to Cortical Glutamate Dynamics in Parkinson's Disease—A 7‐T Functional Magnetic Resonance Spectroscopy Study,” European Journal of Neurology 30, no. 7 (2023): 2106–2111, 10.1111/ene.15818. 37038631

[26] N. T. de Joode , O. A. van den Heuvel , M. Koster , et al., “Glutamate Dynamics and BOLD Response During OCD Symptom Provocation in the Lateral Occipital Cortex: A 7 Tesla fMRI‐fMRS Study,” Journal of Affective Disorders 367 (2024): 416–425, 10.1016/j.jad.2024.08.216. 39233246

[27] P. Sarchielli , R. Tarducci , O. Presciutti , et al., “Functional ^1^H‐MRS Findings in Migraine Patients With and Without Aura Assessed Interictally,” NeuroImage 24, no. 4 (2005): 1025–1031, 10.1016/j.neuroimage.2004.11.005. 15670679

[28] P. S. Sándor , U. Dydak , J. Schoenen , et al., “MR‐Spectroscopic Imaging During Visual Stimulation in Subgroups of Migraine With Aura,” Cephalalgia 25, no. 7 (2005): 507–518, 10.1111/j.1468-2982.2005.00900.x. 15955037

[29] G.‐H. Jahng , J. Oh , D.‐W. Lee , et al., “Glutamine and Glutamate Complex, as Measured by Functional Magnetic Resonance Spectroscopy, Alters During Face‐Name Association Task in Patients With Mild Cognitive Impairment and Alzheimer's Disease,” Journal of Alzheimer's Disease 52, no. 1 (2016): 145–159, 10.3233/jad-150877. 27060946

[30] L. K. Bak , A. Schousboe , and H. S. Waagepetersen , “The Glutamate/GABA‐Glutamine Cycle: Aspects of Transport, Neurotransmitter Homeostasis and Ammonia Transfer,” Journal of Neurochemistry 98, no. 3 (2006): 641–653, 10.1111/j.1471-4159.2006.03913.x. 16787421

[31] J. V. Andersen , “The Glutamate/GABA‐Glutamine Cycle: Insights, Updates, and Advances,” Journal of Neurochemistry 169, no. 3 (2025): e70029, 10.1111/jnc.70029. 40066661 PMC11894596

[32] D. Pasanta , J. L. He , T. Ford , G. Oeltzschner , D. J. Lythgoe , and N. A. Puts , “Functional MRS Studies of GABA and Glutamate/Glx—A Systematic Review and Meta‐Analysis,” Neuroscience and Biobehavioral Reviews 144 (2023): 104940, 10.1016/j.neubiorev.2022.104940. 36332780 PMC9846867

[33] M. Martínez‐Maestro , C. Labadie , and H. E. Möller , “Dynamic Metabolic Changes in Human Visual Cortex in Regions With Positive and Negative Blood Oxygenation Level‐Dependent Response,” Journal of Cerebral Blood Flow and Metabolism: Official Journal of the International Society of Cerebral Blood Flow and Metabolism 39, no. 11 (2019): 2295–2307, 10.1177/0271678x18795426. 30117749 PMC6827122

[34] Y. Boillat , L. Xin , W. van der Zwaag , and R. Gruetter , “Metabolite Concentration Changes Associated With Positive and Negative BOLD Responses in the Human Visual Cortex: A Functional MRS Study at 7 Tesla,” Journal of Cerebral Blood Flow and Metabolism: Official Journal of the International Society of Cerebral Blood Flow and Metabolism 40, no. 3 (2020): 488–500, 10.1177/0271678x19831022. 30755134 PMC7026843

[35] J. Jorge , P. Figueiredo , R. Gruetter , and W. van der Zwaag , “Mapping and Characterization of Positive and Negative BOLD Responses to Visual Stimulation in Multiple Brain Regions at 7T,” Human Brain Mapping 39, no. 6 (2018): 2426–2441, 10.1002/hbm.24012. 29464809 PMC6866646

[36] Y. Hlushchuk and R. Hari , “Transient Suppression of Ipsilateral Primary Somatosensory Cortex During Tactile Finger Stimulation,” Journal of Neuroscience 26, no. 21 (2006): 5819–5824, 10.1523/jneurosci.5536-05.2006. 16723540 PMC6675271

[37] L. Pellerin , “Lactate as a Pivotal Element in Neuron–Glia Metabolic Cooperation,” Neurochemistry International 43, no. 4–5 (2003): 331–338, 10.1016/s0197-0186(03)00020-2. 12742077

[38] M. K. Jha and B. M. Morrison , “Lactate Transporters Mediate Glia‐Neuron Metabolic Crosstalk in Homeostasis and Disease,” Frontiers in Cellular Neuroscience 14 (2020): 589582, 10.3389/fncel.2020.589582. 33132853 PMC7550678

[39] K. H. Lauritzen , C. Morland , M. Puchades , et al., “Lactate Receptor Sites Link Neurotransmission, Neurovascular Coupling, and Brain Energy Metabolism,” Cerebral Cortex 24, no. 10 (2013): 2784–2795, 10.1093/cercor/bht136. 23696276

[40] M. DiNuzzo , G. A. Dienel , K. L. Behar , et al., “Neurovascular Coupling Is Optimized to Compensate for the Increase in Proton Production From Nonoxidative Glycolysis and Glycogenolysis During Brain Activation and Maintain Homeostasis of pH, *p*CO_2_, and *p*O_2_ ,” Journal of Neurochemistry 168, no. 5 (2023): 632–662, 10.1111/jnc.15839. 37150946 PMC10628336

[41] L. Pellerin and P. J. Magistretti , “Glutamate Uptake Into Astrocytes Stimulates Aerobic Glycolysis: A Mechanism Coupling Neuronal Activity to Glucose Utilization,” Proceedings of the National Academy of Sciences of the United States of America 91, no. 22 (1994): 10625–10629, 10.1073/pnas.91.22.10625. 7938003 PMC45074

[42] P. T. Fox , M. E. Raichle , M. A. Mintun , and C. Dence , “Nonoxidaive Glucose Consumption During Focal Physiologic Neural Activity,” Science 241 (1988): 462–464. 3260686 10.1126/science.3260686

[43] I. A. Simpson , A. Carruthers , and S. J. Vannucci , “Supply and Demand in Cerebral Energy Metabolism: The Role of Nutrient Transporters,” Journal of Cerebral Blood Flow and Metabolism: Official Journal of the International Society of Cerebral Blood Flow and Metabolism 27, no. 11 (2007): 1766–1791. 17579656 10.1038/sj.jcbfm.9600521 PMC2094104

[44] M. DiNuzzo , S. Mangia , B. Maraviglia , and F. Giove , “Changes in Glucose Uptake Rather Than Lactate Shuttle Take Center Stage in Subserving Neuroenergetics: Evidence From Mathematical Modeling,” Journal of Cerebral Blood Flow & Metabolism 30, no. 3 (2009): 586–602, 10.1038/jcbfm.2009.232. 19888285 PMC2949148

[45] S. Mangia , M. DiNuzzo , F. Giove , A. Carruthers , I. A. Simpson , and S. J. Vannucci , “Response to ‘Comment on Recent Modeling Studies of Astrocyte—Neuron Metabolic Interactions’: Much Ado About Nothing,” Journal of Cerebral Blood Flow and Metabolism 31, no. 6 (2011): 1346–1353, 10.1038/jcbfm.2011.29. 21427731 PMC3130323

[46] G. A. Dienel , D. L. Rothman , and S. Mangia , “A Bird's‐Eye View of Glycolytic Upregulation in Activated Brain: The Major Fate of Lactate Is Release From Activated Tissue, Not Shuttling to Nearby Neurons,” Journal of Neurochemistry 169, no. 6 (2025): e70111, 10.1111/jnc.70111. 40476345 PMC12142580

[47] J. P. Bolaños , C. M. Alberini , A. Almeida , et al., “Embracing the Modern Biochemistry of Brain Metabolism,” Journal of Neurochemistry 169, no. 7 (2025): e70166, 10.1111/jnc.70166. 40686252

[48] J. V. Andersen , B. I. Aldana , L. K. Bak , et al., “Embracing Scientific Debate in Brain Metabolism,” Journal of Neurochemistry 169, no. 9 (2025): e70230, 10.1111/jnc.70230. 40966093

[49] N. Bueschke , L. Amaral‐Silva , M. Hu , and J. Santin , “Lactate Ions Induce Synaptic Plasticity to Enhance Output From the Central Respiratory Network,” Journal of Physiology 599 (2021): 5485–5504. 34761806 10.1113/JP282062 PMC8696744

[50] C. M. Alberini , E. Cruz , G. Descalzi , B. Bessières , and V. Gao , “Astrocyte Glycogen and Lactate: New Insights Into Learning and Memory Mechanisms,” Glia 66, no. 6 (2017): 1244–1262, 10.1002/glia.23250. 29076603 PMC5903986

[51] L. El Hayek , M. Khalifeh , V. Zibara , et al., “Lactate Mediates the Effects of Exercise on Learning and Memory Through SIRT1‐Dependent Activation of Hippocampal Brain‐Derived Neurotrophic Factor (BDNF),” Journal of Neuroscience 39 (2019): 2369–2382, 10.1523/jneurosci.1661-18.2019. 30692222 PMC6435829

[52] N. Karnib , R. El‐Ghandour , L. El Hayek , et al., “Lactate Is an Antidepressant That Mediates Resilience to Stress by Modulating the Hippocampal Levels and Activity of Histone Deacetylases,” Neuropsychopharmacology 44, no. 6 (2019): 1152–1162, 10.1038/s41386-019-0313-z. 30647450 PMC6461925

[53] A. Tauffenberger , H. Fiumelli , S. Almustafa , and P. J. Magistretti , “Lactate and Pyruvate Promote Oxidative Stress Resistance Through Hormetic ROS Signaling,” Cell Death & Disease 10, no. 9 (2019): 653. 31506428 10.1038/s41419-019-1877-6 PMC6737085

[54] M. S. Vafaee , K. Vang , L. H. Bergersen , and A. Gjedde , “Oxygen Consumption and Blood Flow Coupling in Human Motor Cortex During Intense Finger Tapping: Implication for a Role of Lactate,” Journal of Cerebral Blood Flow and Metabolism 32, no. 10 (2012): 1859–1868, 10.1038/jcbfm.2012.89. 22781333 PMC3463880

[55] L. H. Bergersen and A. Gjedde , “Is Lactate a Volume Transmitter of Metabolic States of the Brain?,” Frontiers in Neuroenergetics 4 (2012): 5, 10.3389/fnene.2012.00005. 22457647 PMC3307048

[56] D. Sappey‐Marinier , G. Calabrese , G. Fein , J. W. Hugg , C. Biggins , and M. W. Weiner , “Effect of Photic Stimulation on Human Visual Cortex Lactate and Phosphates Using ^1^H and ^31^P Magnetic Resonance Spectroscopy,” Journal of Cerebral Blood Flow and Metabolism 12 (1992): 584–592. 1618937 10.1038/jcbfm.1992.82

[57] J. Frahm , G. Kruger , K. Merboldt , and A. Kleinschmidt , “Dynamic Uncoupling and Recoupling of Perfusion and Oxidative Metabolism During Focal Brain Activation in Man,” Magnetic Resonance in Medicine 35 (1996): 143–148. 8622575 10.1002/mrm.1910350202

[58] P. G. Mullins , “Towards a Theory of Functional Magnetic Resonance Spectroscopy (fMRS): A Meta‐Analysis and Discussion of Using MRS to Measure Changes in Neurotransmitters in Real Time,” Scandinavian Journal of Psychology 59, no. 1 (2018): 91–103, 10.1111/sjop.12411. 29356002

[59] A. M. Wang , G. K. K. Leung , K. M. Y. Kiang , D. Chan , P. Cao , and E. X. Wu , “Separation and Quantification of Lactate and Lipid at 1.3 ppm by Diffusion‐Weighted Magnetic Resonance Spectroscopy,” Magnetic Resonance in Medicine 77, no. 2 (2016): 480–489, 10.1002/mrm.26144. 26833380

[60] M. Wilson , O. Andronesi , P. B. Barker , et al., “Methodological Consensus on Clinical Proton MRS of the Brain: Review and Recommendations,” Magnetic Resonance in Medicine 82, no. 2 (2019): 527–550, 10.1002/mrm.27742. 30919510 PMC7179569

[61] A.‐L. Lin , P. T. Fox , J. Hardies , T. Q. Duong , and J.‐H. Gao , “Nonlinear Coupling Between Cerebral Blood Flow, Oxygen Consumption, and ATP Production in Human Visual Cortex,” National Academy of Sciences of the United States of America 107, no. 18 (2010): 8446–8451, 10.1073/pnas.0909711107. PMC2889577 20404151

[62] S. J. Nichols , J. A. Yanes , M. A. Reid , and J. L. Robinson , “7 T Characterization of Excitatory and Inhibitory Systems of Acute Pain in Healthy Female Participants,” NMR in Biomedicine 37, no. 4 (2023): e5088, 10.1002/nbm.5088. 38140895

[63] Y. Koush , R. A. de Graaf , R. Kupers , et al., “Metabolic Underpinnings of Activated and Deactivated Cortical Areas in Human Brain,” Journal of Cerebral Blood Flow and Metabolism 41, no. 5 (2021): 986–1000, 10.1177/0271678x21989186. 33472521 PMC8054719

[64] R. S. Koolschijn , W. T. Clarke , I. B. Ip , U. E. Emir , and H. C. Barron , “Event‐Related Functional Magnetic Resonance Spectroscopy,” NeuroImage 276 (2023): 120194, 10.1016/j.neuroimage.2023.120194. 37244321 PMC7614684

[65] S. Mangia , G. Garreffa , M. Bianciardi , F. Giove , F. Di Salle , and B. Maraviglia , “The Aerobic Brain: Lactate Decrease at the Onset of Neural Activity,” Neuroscience 118, no. 1 (2003): 7–10, 10.1016/s0306-4522(02)00792-3. 12676131

[66] M. J. Page , J. E. McKenzie , P. M. Bossuyt , et al., “Statement: An Updated Guideline for Reporting Systematic Reviews,” BMJ 2021 (2020): n71, 10.1136/bmj.n71. PMC8005924 33782057

[67] Y. Koush , D. L. Rothman , K. L. Behar , R. A. de Graaf , and F. Hyder , “Human Brain Functional MRS Reveals Interplay of Metabolites Implicated in Neurotransmission and Neuroenergetics,” Journal of Cerebral Blood Flow and Metabolism 42, no. 6 (2022): 911–934, 10.1177/0271678x221076570. 35078383 PMC9125492

[68] A. S. Urrila , A. Hakkarainen , S. Heikkinen , et al., “Stimulus‐Induced Brain Lactate: Effects of Aging and Prolonged Wakefulness,” Journal of Sleep Research 13, no. 2 (2004): 111–119, 10.1111/j.1365-2869.2004.00401.x. 15175090

[69] J. Frahm , K. D. Merboldt , and W. Hänicke , “Localized Proton Spectroscopy Using Stimulated Echoes,” Journal of Magnetic Resonance 72, no. 3 (1987): 502–508.

[70] P. A. Bottomley , “Spatial Localization in NMR Spectroscopy In Vivo,” Annals of the New York Academy of Sciences 508 (1987): 333–348, 10.1111/j.1749-6632.1987.tb32915.x. 3326459

[71] G. Öz and I. Tkáč , “Short‐Echo, Single‐Shot, Full‐Intensity Proton Magnetic Resonance Spectroscopy for Neurochemical Profiling at 4 T: Validation in the Cerebellum and Brainstem,” Magnetic Resonance in Medicine 65, no. 4 (2010): 901–910, 10.1002/mrm.22708. 21413056 PMC3827699

[72] V. Mlynárik , G. Gambarota , H. Frenkel , and R. Gruetter , “Localized Short‐Echo‐Time Proton MR Spectroscopy With Full Signal‐Intensity Acquisition,” Magnetic Resonance in Medicine 56, no. 5 (2006): 965–970, 10.1002/mrm.21043. 16991116

[73] T. W. H. Kuwabara , S. Tsuji , and T. Yuasa , “Lactate Rise in the Basal Ganglia Accompanying Finger Movements: A Localized ^1^H‐MRS Study,” Brain Research 670 (1995): 326–328. 7743199 10.1016/0006-8993(94)01353-j

[74] A. S. Urrila , A. Hakkarainen , S. Heikkinen , et al., “Metabolic Imaging of Human Cognition: An fMRI/^1^H‐MRS Study of Brain Lactate Response to Silent Word Generation,” Journal of Cerebral Blood Flow and Metabolism 23, no. 8 (2003): 942–948, 10.1097/01.wcb.0000080652.64357.1d. 12902838

[75] S. Mangia , I. Tkác̆ , N. K. Logothetis , R. Gruetter , P. F. Van de Moortele , and K. Uğurbil , “Dynamics of Lactate Concentration and Blood Oxygen Level‐Dependent Effect in the Human Visual Cortex During Repeated Identical Stimuli,” Journal of Neuroscience Research 85, no. 15 (2007): 3340–3346, 10.1002/jnr.21371. 17526022

[76] Y. Lin , M. C. Stephenson , L. Xin , A. Napolitano , and P. G. Morris , “Investigating the Metabolic Changes due to Visual Stimulation Using Functional Proton Magnetic Resonance Spectroscopy at 7 T,” Journal of Cerebral Blood Flow and Metabolism: Official Journal of the International Society of Cerebral Blood Flow and Metabolism 32, no. 8 (2012): 1484–1495, 10.1038/jcbfm.2012.33. 22434070 PMC3421086

[77] T.‐H. Kim , H.‐K. Kang , and G.‐W. Jeong , “Assessment of Brain Metabolites Change During Visual Sexual Stimulation in Healthy Women Using Functional MR Spectroscopy,” Journal of Sexual Medicine 10, no. 4 (2013): 1001–1011, 10.1111/jsm.12057. 23347356

[78] B. Schaller , R. Mekle , L. Xin , N. Kunz , and R. Gruetter , “Net Increase of Lactate and Glutamate Concentration in Activated Human Visual Cortex Detected With Magnetic Resonance Spectroscopy at 7 Tesla,” Journal of Neuroscience Research 91, no. 8 (2013): 1076–1083, 10.1002/jnr.23194. 23378234

[79] P. Bednařík , I. Tkáč , F. Giove , et al., “Neurochemical and BOLD Responses During Neuronal Activation Measured in the Human Visual Cortex at 7 Tesla,” Journal of Cerebral Blood Flow and Metabolism: Official Journal of the International Society of Cerebral Blood Flow and Metabolism 35, no. 4 (2015): 601–610, 10.1038/jcbfm.2014.233. 25564236 PMC4420878

[80] R. Mekle , S. Kühn , H. Pfeiffer , S. Aydin , F. Schubert , and B. Ittermann , “Detection of Metabolite Changes in Response to a Varying Visual Stimulation Paradigm Using Short‐TE ^1^H MRS at 7 T,” NMR in Biomedicine 30, no. 2 (2017): e3672, 10.1002/nbm.3672. 28008663

[81] P. Bednařík , I. Tkáč , F. Giove , et al., “Neurochemical Responses to Chromatic and Achromatic Stimuli in the Human Visual Cortex,” Journal of Cerebral Blood Flow and Metabolism: Official Journal of the International Society of Cerebral Blood Flow and Metabolism 38, no. 2 (2018): 347–359, 10.1177/0271678x17695291. 28273721 PMC5951013

[82] Y. Koush , R. A. de Graaf , L. Jiang , D. L. Rothman , and F. Hyder , “Functional MRS With J‐Edited Lactate in Human Motor Cortex at 4 T,” NeuroImage 184 (2019): 101–108, 10.1016/j.neuroimage.2018.09.008. 30201463

[83] C. C. Fernandes , B. Lanz , C. Chen , and P. G. Morris , “Measurement of Brain Lactate During Visual Stimulation Using a Long TE Semi‐LASER Sequence at 7 T,” NMR in Biomedicine 33, no. 4 (2020): e4223, 10.1002/nbm.4223. 31995265 PMC7079106

[84] F. T. van Vugt , J. Near , T. Hennessy , J. Doyon , and D. J. Ostry , “Early Stages of Sensorimotor Map Acquisition: Neurochemical Signature in Primary Motor Cortex and Its Relation to Functional Connectivity,” Journal of Neurophysiology 124 (2020): 1615–1624. 32997558 10.1152/jn.00285.2020 PMC7814893

[85] M. DiNuzzo , S. Mangia , F. Giove , G. E. Hagberg , D. Mascali , and M. Moraschi , “Perception Is Associated With the Brain's Metabolic Response to Sensory Stimulation,” eLife 11 (2022): e71016, 10.7554/eLife.71016. 35225790 PMC9038191

[86] J. Dorst , T. Borbath , K. Landheer , N. Avdievich , and A. Henning , “Simultaneous Detection of Metabolite Concentration Changes, Water BOLD Signal and pH Changes During Visual Stimulation in the Human Brain at 9.4T,” Journal of Cerebral Blood Flow and Metabolism: Official Journal of the International Society of Cerebral Blood Flow and Metabolism 42, no. 6 (Jun 2022): 1104–1119, 10.1177/0271678x221075892. 35060409 PMC9121534

[87] M. Morelli , K. Dudzikowska , D. K. Deelchand , et al., “Functional Magnetic Resonance Spectroscopy of Prolonged Motor Activation Using Conventional and Spectral GLM Analyses,” Imaging Neuroscience 3 (2025): imag_a_00452, 10.1162/imag_a_00452. 40800844 PMC12319857

[88] F. Träber , W. Block , R. Lamerichs , J. Gieseke , and H. H. Schild , “ ^1^H Metabolite Relaxation Times at 3.0 Tesla: Measurements of T1 and T2 Values in Normal Brain and Determination of Regional Differences in Transverse Relaxation,” Journal of Magnetic Resonance Imaging 19, no. 5 (2004): 537–545, 10.1002/jmri.20053. 15112302

[89] P. O. Wyss , C. Bianchini , M. Scheidegger , et al., “In Vivo Estimation of Transverse Relaxation Time Constant (T2) of 17 Human Brain Metabolites at 3T,” Magnetic Resonance in Medicine 80, no. 2 (2018): 452–461, 10.1002/mrm.27067. 29344979

[90] L. Jouvensal , P. G. Carlier , and G. Bloch , “Evidence for Bi‐Exponential Transverse Relaxation of Lactate in Excised Rat Muscle,” Magnetic Resonance in Medicine 41, no. 3 (1999): 624–626, 10.1002/(sici)1522-2594(199903)41:3<624::aid-mrm27>3.0.co;2-w. 10204888

[91] S. Murali‐Manohar , T. Borbath , A. M. Wright , B. Soher , R. Mekle , and A. Henning , “T2 Relaxation Times of Macromolecules and Metabolites in the Human Brain at 9.4 T,” Magnetic Resonance in Medicine 84, no. 2 (2020): 542–558, 10.1002/mrm.28174. 32003506

[92] F. F. Paiva , M. C. G. Otaduy , R. de Oliveira‐Souza , et al., “Comparison of Human Brain Metabolite Levels Using ^1^H MRS at 1.5T and 3.0T,” Dementia & Neuropsychologia 7, no. 2 (2013): 216–220. 29213843 10.1590/S1980-57642013DN70200013 PMC5619521

[93] M. Terpstra , I. Cheong , T. Lyu , et al., “Test‐Retest Reproducibility of Neurochemical Profiles With Short‐Echo, Single‐Voxel MR Spectroscopy at 3T and 7T,” Magnetic Resonance in Medicine 76, no. 4 (2015): 1083–1091, 10.1002/mrm.26022. 26502373 PMC4846596

[94] D. K. Deelchand , I. Iltis , and P.‐G. Henry , “Improved Quantification Precision of Human Brain Short Echo‐Time ^1^H Magnetic Resonance Spectroscopy at High Magnetic Field: A Simulation Study,” Magnetic Resonance in Medicine 72, no. 1 (2014): 20–25, 10.1002/mrm.24892. 23900976 PMC3907456

[95] C. E. Landisman and D. Y. Ts'O , “Color Processing in Macaque Striate Cortex: Electrophysiological Properties,” Journal of Neurophysiology 87 (2002): 3138–3151. 12037214 10.1152/jn.00957.1999

[96] H. D. Lu and A. W. Roe , “Functional Organization of Color Domains in V1 and V2 of Macaque Monkey Revealed by Optical Imaging,” Cerebral Cortex 18, no. 3 (2007): 516–533, 10.1093/cercor/bhm081. 17576751 PMC2657473

[97] M. S. Vafaee and A. Gjedde , “Model of Blood–Brain Transfer of Oxygen Explains Nonlinear Flow‐Metabolism Coupling During Stimulation of Visual Cortex,” Journal of Cerebral Blood Flow & Metabolism 20, no. 4 (2000): 747–754. 10779019 10.1097/00004647-200004000-00012

[98] A. L. Lin , P. T. Fox , Y. Yang , H. Lu , L. H. Tan , and J. H. Gao , “Evaluation of MRI Models in the Measurement of CMRO2 and Its Relationship With CBF,” Magnetic Resonance in Medicine 60, no. 2 (2008): 380–389, 10.1002/mrm.21655. 18666102 PMC2612533

[99] S. Sonnay , J. M. N. Duarte , and N. Just , “Lactate and Glutamate Dynamics During Prolonged Stimulation of the Rat Barrel Cortex Suggest Adaptation of Cerebral Glucose and Oxygen Metabolism,” Neuroscience 346 (2017): 337–348, 10.1016/j.neuroscience.2017.01.034. 28153690

[100] R. B. Buxton , K. Uludağ , D. J. Dubowitz , and T. T. Liu , “Modeling the Hemodynamic Response to Brain Activation,” NeuroImage 23 (2004): S220–S233, 10.1016/j.neuroimage.2004.07.013. 15501093

[101] N. K. Logothetis , J. Pauls , M. Augath , T. Trinath , and A. Oeltermann , “Neurophysiological Investigation of the Basis of the fMRI Signal,” Nature 412 (2001): 150–157. 11449264 10.1038/35084005

[102] X. H. Zhu and W. Chen , “Observed BOLD Effects on Cerebral Metabolite Resonances in Human Visual Cortex During Visual Stimulation: A Functional ^1^H MRS Study at 4 T,” Magnetic Resonance in Medicine 46, no. 5 (2001): 841–847, 10.1002/mrm.1267. 11675633

[103] M. A. Macrì , G. Garreffa , F. Giove , et al., “In Vivo Quantitative ^1^H MRS of Cerebellum and Evaluation of Quantitation Reproducibility by Simulation of Different Levels of Noise and Spectral Resolution,” Magnetic Resonance Imaging 22, no. 10 (2004): 1385–1393, 10.1016/j.mri.2004.10.021. 15707788

