# Supplementary material for: Lactate Changes Quantified by fMRS: A Meta‐Analysis
Source: NMR Biomed. 2026 May 4;39:e70295. doi: 10.1002/nbm.70295 (PMC13137767; doi:10.1002/nbm.70295)
Supplement: Supplementary file 1 — Figure S1: Funnel plot of the studies included in the meta‐analysis. Each study is color‐coded by year of publication. Figure S2: Scatter plot with effect size and block stimulation duration. [file NBM-39-e70295-s001.docx]

Lactate changes quantified by fMRS: a meta-analysis

Luca Cairone^1,2^, Maria Guidi^2^, Matteo Mancini^2,3^, Federico Giove^2,4^*

*^1^Università degli Studi di Roma La Sapienza, Rome, Italy*

*^2^Centro Ricerche Enrico Fermi, Rome, Italy*

*^3^Cardiff University Brain Research Imaging Centre (CUBRIC), Cardiff University, Cardiff, United Kingdom*

*^4^Fondazione Santa Lucia IRCCS, Rome, Italy*

**SUPPLEMENTARY MATERIAL**

Figure 1S shows the funnel plot displaying the effect sizes of the studies included in the meta-analysis as a function of their standard errors (SE). Each study is color-coded according to its year of publication. Although some older studies exhibit relatively larger SEs, this pattern is not consistent, as several older studies are also located in the upper portion of the funnel plot. More recent studies tend to cluster closer to the top of the funnel, reflecting higher precision, likely due to larger sample sizes or methodological improvements. The non-significant p-value obtained from the Egger test (p=0.34) suggests no evidence of publication bias.


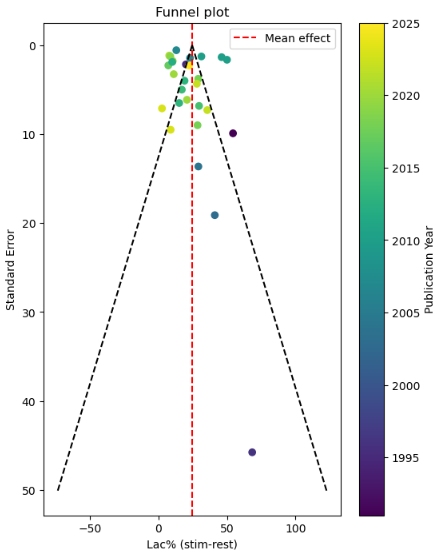


Figure 1S Funnel plot of the studies included in the meta-analysis. Each study is color-coded by year of publication.

Figure 2S Scatter plot with effect size and block stimulation duration
